# Effects of vonoprazan on gastric PH and clinical course after gastric ESD: A retrospective and prospective study

**DOI:** 10.1016/j.amsu.2020.10.002

**Published:** 2020-10-07

**Authors:** Daisuke Suto, Masashi Yoshida, Takaaki Otake, Eiichiro Ichiishi, Kiichi Sato, Kazumoto Murata, Hirotoshi Ebinuma, Hironori Odaira, Yutaka Suzuki, Yutaka Kohgo

**Affiliations:** a Department of Gastroenterology, International University of Health and Welfare Hospital, 537-3 Iguchi, Nasushiobara, Tochigi, 329-2763, Japan; b Department of Surgery, International University of Health and Welfare Hospital, 537-3, Iguchi, Nasushiobara, Tochigi, 329-2763, Japan; c Department of Gastroenterology and Hepatology, International University of Health and Welfare, Narita Hospital, 852, Hatagata, Narita, Chiba, 286-8520, Japan

**Keywords:** Vonoprazan, Endoscopic submucosal dissection, pH, Ulcer, ESD, endoscopic submucosal dissection, PPI, proton-pump inhibitor

## Abstract

**Background:**

Vonoprazan has been more widely used for artificial ulcers after endoscopic submucosal dissection (ESD) for early gastric cancer; however, no reports have examined intragastric pH during ESD. The present study aimed to measure gastric pH at the time of ESD and the clinical course afterwards for patients treated with vonoprazan the night before undergoing ESD.

**Materials and methods:**

We examined medication status regarding gastric acid secretion and antithrombotic drugs, post-ESD bleeding as a perioperative complication, and the timing of upper gastrointestinal endoscopy after ESD and ulcer healing in 156 patients who underwent gastric ESD at our hospital from January 2014 to December 2019. The gastric pH was measured at the time of ESD after administration of 20 mg vonoprazan on the night before gastric ESD.

**Results:**

There were 14 cases of post-ESD bleeding in patients treated with proton-pump inhibitors (PPIs), including oozing during second-look endoscopy compared to only 1 case of bleeding with vonoprazan administration (p < 0.05). Vonoprazan was also associated with better post-ESD ulcer healing than PPIs. Gastric pH during ESD after vonoprazan administration on the night before gastric ESD was ≥6.96 in all 11 patients.

**Conclusion:**

Post-ESD bleeding was reduced, and ulcer healing was improved in patients treated with vonoprazan the night before their procedure. Our results suggest high gastric pH during ESD due to vonoprazan administration may be beneficial for hemostasis and ulcer healing following ESD.

## Highlights

• Post-ESD bleeding was reduced vonoprazan treated in patient.
• Gastric pH at ESD was high after vonoprazan treatment on the night before ESD.
• Post-ESD ulcer healing was improved vonoprazan treated in patient.

## 1 Introduction

Several studies have reported on the effects of vonoprazan on post-endoscopic submucosal dissection (ESD) ulcers; however, none have reported the effects of gastric pH during ESD. It takes several days for the gastric pH to reach a high level with conventional gastric acid secretion inhibitors, such as proton-pump inhibitors (PPIs) [1]. Vonoprazan, a potassium-competitive acid blocker [2] that has been available since February 2015, is highly basic, accumulates in the secretory tubules of parietal cells for a long time [3], and is more potent than PPIs [4]. Gastric pH reportedly rises to ≥4 within 4 h after vonoprazan administration [3,5]. ESD is performed as an endoscopic treatment for early gastric cancer; however, the incidence of bleeding or perforation from an artificial ulcer after ESD has been reported as approximately 3.5% [[6], [7], [8], [9], [10]].

PPI administration after ESD suppresses gastric acid secretion and improves post-ESD ulcer healing, especially because gastric acid is involved in the development of post-ESD bleeding [[11], [12], [13]]. However, even if PPIs are administered after ESD, there is a risk of bleeding because PPIs cannot adequately increase gastric pH on the day of or day after ESD [3,5]. Therefore, second-look endoscopy and clinical findings on the day after ESD are important. Vonoprazan has been reported to increase intragastric pH immediately after oral administration [3,5]; therefore, its administration on the day before ESD should increase intragastric pH during ESD and may thereby reduce the risk of bleeding. Vonoprazan is now popular for treating artificial ulcers occurring after ESD performed for early gastric cancer and is superior to PPIs in preventing post-ESD bleeding [14,15] and promoting ulcer healing [16,17]. Although gastric pH is considered important for ulcer healing and preventing upper gastrointestinal bleeding, the effects of vonoprazan administration on gastric pH at the time of ESD have not been reported. In our hospital, a second-look endoscopy is often performed the day after ESD. Therefore, the present study aimed to examine the actual clinical course (including findings from the second-look endoscopy coupled with the clinical assessment) and measure gastric pH at the time of ESD after vonoprazan administration on the night before gastric ESD.

## 2 Material and methods

### 2.1 Retrospective clinical course analysis

From January 2014 to December 2019, 156 patients (115 men and 41 women) who underwent gastric ESD at our hospital were enrolled. Post-ESD course and upper gastrointestinal endoscopy findings were examined from clinical records. The work has been reported in line with the STROCSS criteria [18].

### 2.2 Prospective analysis of intragastric pH

Of the 19 patients who underwent gastric ESD between June 2019 and March 2020, we excluded 5 patients who were taking acid secretion inhibitors, 1 patient who did not provide informed consent, and 2 patients who had no gastric juice in their stomachs. Finally, 11 patients (7 men and 4 women) were included in the present study. Patients were administered 20 mg vonoprazan on the night before gastric ESD. The gastric juice was collected immediately after endoscope insertion in the stomach. The pH meter 206-pH5 (Tesuto co., Kanagawa, Japan) was used. Statistical analysis was performed through the chi-square test using js-STAR version 9.8.3j (http://www.kisnet.or.jp/nappa/software/star/freq/chisq_ixj.htm#).

## 3 Results

### 3.1 Retrospective clinical course analysis

The patients had an average age of 70.7 (45–90) years. Gastric lesions were present in 31 U regions, 68 M regions, and 57 L regions, according to the Japanese classification of gastric cancer [19] (Table 1). Clinical bleeding occurred in 5 patients (3.2%), and minor oozing and bleeding during the second-look endoscopy were noted in 10 patients (6.4%) (Table 2). Vonoprazan administration, since its launch in February 2015, has been increasing annually. From 2014 to 2016, 90 patients (95%, 90/95) used PPIs and 5 patients (5%, 5/95) used vonoprazan. After 2017, vonoprazan use increased to 82% (50/61 patients) compared to PPI use [18% (11/61 patients)] (Table 3).

**Table 1:** Characteristics of patients analyzed in the present study.

Characteristic	Male (n = 115)	Female (n = 41)	Total (n = 156)
Age	46-86 (70.9)	45-90 (70.2)	45-90 (70.7)
Tumor location			
U	24	7	31
M	56	12	68
L	35	22	57
Tumor histopathology			
No tumor	6	3	9
Adenoma	16	12	28
Differentiated carcinoma	92	24	116
Undifferentiated carcinoma	1	2	3
Complications			
Delayed bleeding			
(Hematemesis)	3	2	5 (3.2%)
(Oozing)	8	2	10 (6.4%)
Perforation	4	0	4 (3%)
Anticoagulants	18	6	24

**Table 2:** Post-ESD bleeding rates.

Characteristic		Cases		
Clinical bleeding	Hematemesis	3	5 (3.2%)	15 (9.6%)
	Melena	2		
Bleeding on second-look endoscopy	Minor oozing	2	10 (6.4%)	
	Oozing	6		
	Bleeding	2

**Table 3:** Acid-secretory drug use, number of ESD complications, and antithrombotic drug use by year.

Characteristic	2014 y	2015 y	2016 y	2014–2016 y	2017 y	2018 y	2019 y	2017–2019 y
ESD cases	26	40	29	**95**	27	15	19	**61**
Vonoprazan	0	1 (3%)	4 (14%)	**5 (5%)**	24 (89%)	10 (67%)	16 (84%)	**50 (82%)**
Esomeprazole	25 (96%)	33 (82%)	21 (72%)	**79 (83%)**	3 (11%)	5 (33%)	1 (5%)	**9 (15%)**
Rabeprazole	1 (4%)	6 (15%)	4 (14%)	**11 (12%)**	0	0	2 (11%)	**2 (3%)**
Bleeding	2 (8%)	5 (12.5%)	7 (24%)	14 (15%) *	1 (4%)	0	0	1 (4%) *
Perforation	1 (4%)	2 (5%)	1 (3%)	**4 (4%)**	0	0	0	**0**
Anticoagulants	5	9	5	**19**	2	3	2	**7**
Hemorrhages in patients taking anticoagulants	1 (4%)	4 (10%)	2 (7%)	**7 (7%)**	1 (4%)	0	0	**1 (2%)**

* chi-square test p < 0.05.

Five (3.2%) of the 156 patients experienced clinically problematic bleeding. Of the 90 patients who underwent second-look endoscopy, 10 (11.1%) had minor bleeding during the endoscopy: 8 had minor oozing, and 2 had bleeding (Table 2). PPIs were administered in 14 of 15 patients with bleeding, while only 1 patient was administered vonoprazan (Table 3), who was gastrectomized and experienced bleeding in the residual stomach (Table 4). The bleeding ratio was significantly lower in vonoprazan administered patients. The incidence of bleeding (major and minor) was 13.8% (14/101) in PPI-administered patients and 2.0% (1/55) in vonoprazan-administered patients.

**Table 4:** Number of cases with bleeding (including oozing) in the first three years and the second three years of ESD.

Characteristic	2014-2016y	2017-2019y
Bleeding Cases	14	1 (residual stomach)
Vonoprazan	0	1
Esomeprazole	11	0
Rabeprazole	3	0
Anticoagulants	6	1
Kimura-takemoto Classification open type	9	1
Tumor location		1
U	2	
M	7	
L	5	

Upper gastrointestinal endoscopy was performed from 51 to 79 (median: 61) days after ESD. Only one patient (3%, 1/32) had an open ulcer among vonoprazan-administered patients. Eleven patients (17%, 11/64) had open ulcers among PPI-administered patients (p < 0.05) (Table 5).

**Table 5:** Status of artificial ulcer healing with vonoprazan or PPI at about 2 months after ESD.

Post-ESD state	Acid secretion inhibitor	
	Vonoprazan	PPI
		Esomeprazole + Rabeprazole
Scar	31	53
Open ulcer	1*	11*

PPI: proton pump inhibitor; ESD: endoscopic submucosal dissection.

*chi-square test p < 0.05.

### 3.2 Prospective analysis on intragastric pH

The average gastric pH was 7.68 ± 0.62 (Table 6). The gastric pH was ≥6.96 in all 11 patients, regardless of the tumor location, histological type, and sex.

**Table 6:** Gastric pH at the time of ESD after vonoprazan administration on the night before ESD.

Characteristic	Male (n = 7)	Female (n = 4)	Total (n = 11)
Age	68-78 (73.4)	51-79 (62)	51-79 (69.3)
Tumor location			
U	0	1	1
M	4	2	6
L	3	1	4
Tumor histopathology			
Adenoma	2	1	3
Differentiated	5	3	8
Undifferentiated	0	0	0
Gastric pH	6.96–8.85 (7.79 ± 0.74(SD))	7.05–7.89 (7.49 ± 0.35(SD))	6.96–8.85 (7.68 ± 0.62(SD))

## 4 Discussion

The present study showed that gastric pH was adequately high under vonoprazan 20 mg administration on the night before gastric ESD, which may be beneficial for hemostasis and healing of ulcers following ESD. When the gastric pH is below 5.4, neither platelet aggregation nor plasma coagulation occurs [20]. Because blood coagulation and platelet aggregation are pH-dependent [13], suppression of gastric acid is important for the prevention of gastric bleeding [[21], [22], [23]].

A high intragastric pH at the time of ESD would be advantageous for hemostasis during surgery and prevention of bleeding immediately after ESD. In this study, 14 of 101 PPI-administered patients (13.8%) experienced bleeding compared to only 1 (2%) of 55 vonoprazan-administered patients, including subclinical bleeding cases during second-look endoscopy.

Vonoprazan is a very powerful acid suppresser [2,14,24], and post-ESD bleeding has been reported to be approximately 3.5% [[6], [7], [8], [9], [10]]. Vonoprazan effectively prevents gastric ulcer bleeding after ESD [15] and has a lower bleeding rate after ESD than PPIs [11].

Previous reports have shown that ordinal PPIs delay sustained gastric acid secretion [25]; however, vonoprazan can reach steady-state acid levels early after intake [1]. Most studies report that vonoprazan is better at healing ulcers than PPIs. Tsuchiya et al. [16] and Maruoka et al. [17] reported that the rate of ulcer scarring after ESD was significantly higher with vonoprazan than with esomeprazole. Vonoprazan is a potent gastric acid secretion inhibitor and effective in healing ulcers after gastric ESD. However, Miwa et al. [26] and Takahashi et al. [27] reported that ulcer healing after ESD was not significantly different between vonoprazan and PPI. This difference in the results has been attributed to the different sizes of the ulcers after ESD and the number and background of the cases. In our study, only one vonoprazan-administered patient (1/32, 3%) did not have ulcer scarring during upper gastrointestinal endoscopy performed about 8 weeks after ESD. In contrast, 11 PPI-administered patients (11/64, 17%) had open ulcers. The scarring ratio was significantly higher in the vonoprazan administered patients. Limitations of this study include it being conducted a single-center study with relatively small sample size; however, it is thought that increasing stomach pH early by vonoprazan administration will have an advantageous effect on ulcer healing. Thus, vonoprazan administration on the day before ESD may contribute to the suppression of bleeding during and after ESD.

Preoperative administration of vonoprazan before the ESD may reduce the risk of bleeding and make post-ESD management even more comfortable.

## 5 Conclusions

Our study showed that gastric pH is adequately high after vonoprazan administration on the day before ESD, which presumably facilitates hemostasis and healing of ulcers following ESD. With preoperative vonoprazan administration, post-ESD bleeding was less frequent, and post-ESD ulcer healing was better.

## Ethical approval

The study was approved by the Ethics Committee of International University of Health and Welfare Hospital [approval number: 13-B-355]. The study participants have given oral and written consent.

## Sources of funding

None to declare.

## Author contribution

All authors contributed equally to the manuscript.

## Research registration number

The study protocol was registered at UMIN Clinical Trials Registry (https://upload.umin.ac.jp/cgi-open-bin/ctr_e/index.cgi), registration number UMIN000041299.

## Guarantor

Daisuke Suto, First Author.

Masashi Yoshida, Senior Author.

## Provenance and peer review

Not commissioned, externally peer reviewed.

## Consent

All patients provided written informed consent.

## Declaration of competing interest

None.
